# Endoplasmic reticulum stress: an emerging therapeutic target for preeclampsia^†^

**DOI:** 10.1093/biolre/ioaf071

**Published:** 2025-03-28

**Authors:** Mengting Chen, Yafang Jin, Xiaohui Cao

**Affiliations:** Department of Obstetrics, Women's Hospital of Jiangnan University, Wuxi Maternity and Child Health Care Hospital, No. 48, Samuel Lane, Liangxi District, Wuxi 214002, China; Department of Obstetrics, Women's Hospital of Jiangnan University, Wuxi Maternity and Child Health Care Hospital, No. 48, Samuel Lane, Liangxi District, Wuxi 214002, China; Department of Obstetrics, Women's Hospital of Jiangnan University, Wuxi Maternity and Child Health Care Hospital, No. 48, Samuel Lane, Liangxi District, Wuxi 214002, China

**Keywords:** preeclampsia, endoplasmic reticulum stress, inflammation, apoptosis, pregnancy, oxidative stress

## Abstract

Preeclampsia (PE), a common obstetric complication during pregnancy, has a global prevalence of 5–8%, and it is one of the leading causes of adverse maternal and fetal outcomes, which has a lack of effective treatment other than termination of pregnancy. Endoplasmic reticulum stress (ERS) is a self-stress response of cells to alleviate misfolded and unfolded protein aggregation and calcium ion homeostasis disorders in the endoplasmic reticulum (ER) lumen by activating the unfolded protein response. Many studies have demonstrated a potential link between ERS and PE pathogenesis by mediating genetic susceptibility, placental hypoxia, oxidative stress, metabolic disorders, impaired angiogenesis and function, and inflammatory responses. This article systematically describes the ERS mechanisms and their association with the pathological progression of PE. It also emphasizes that ERS can be a potential therapeutic target for PE clinical management.

**Highlights:**

- ERS promotes PE development.
- ERS interferes trophoblast apoptosis and trophoblast differentiation.
- ERS stimulates placental hypoxia, metabolic disorders, inflammation, and oxidative stress.
- ERS becomes a target for PE treatment.

## Graphical Abstract

**Graphical Abstract:**
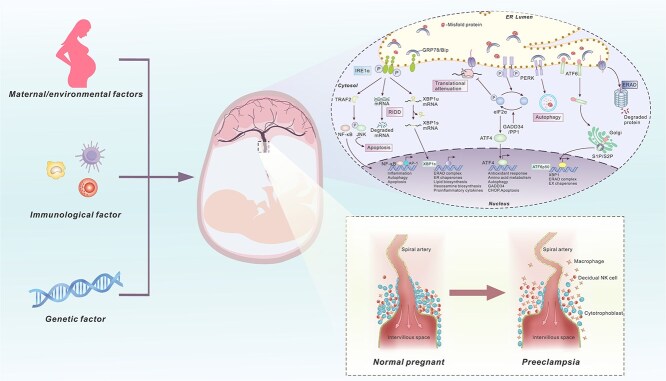

## Introduction

Preeclampsia (PE), as a serious pregnancy complication, poses a significant clinical risk to both the mother and fetus [1]. PE can lead to an increased incidence of neonatal death, fetal growth restriction, fetal distress, and preterm delivery. It is an important cause of maternal and neonatal death, becoming a major public health concern affecting women’s health. A survey found that the global prevalence of PE is about 5–8%, and it is responsible for >500 000 neonatal and >70 000 maternal deaths per year worldwide [2]. It is well known that PE is characterized by proteinuria and hypertension in the middle and later stages of pregnancy and has the potential to rapidly progress to systemic multiorgan damage. PE is divided into two categories: early-onset PE and late-onset PE. Early-onset PE happens before 34 weeks of pregnancy, and it is typically linked to irregular blood flow in the umbilical and uterine arteries, raising the likelihood of complications for both the mother and fetus. Late-onset PE occurs at 34 weeks of gestation or later, with symptoms that are often mild and can be easily missed, resulting in negative perinatal outcomes [3, 4]. Research indicates that early-onset eclampsia occurs less frequently than late-onset eclampsia, yet it has a more significant genetic influence. While the condition is recognized as polygenic [5, 6], the specific molecular mechanisms of inheritance remain unclear. Although the causes and mechanisms underlying PE development remain unclear, numerous studies have shown that the pathologic development of PE can be attributed to a variety of factors, such as genetic factors [7, 8], placental hypoxia [9], endoplasmic reticulum stress (ERS) [10], oxidative stress (OS) [11], metabolic disorders [12], impaired angiogenesis and function [13], and inflammatory responses [14].

PE is a complex pathological process where the abnormal function of the placenta is a key component [15, 16], and it is caused by the interaction between the maternal gestational environment and the embryonic development process [17]. A significant challenge for researchers is determining how to identify the pathological progression of PE, particularly in its early stages when clinical symptoms are absent and diagnosis becomes difficult. Furthermore, there is a shortage of animal models that effectively represent the pathophysiological features of human PE. Currently, clinical therapeutic approaches to PE remain greatly limited due to the lack of understanding of the underlying molecular mechanisms. Medications to control blood pressure and relieve spasms can only prevent pregnancy complications, but the most effective approach remains timely immediate delivery [18, 19]. Various in vivo and ex vivo studies involving molecular, cellular, and tissue levels have confirmed that multiple signaling pathways mediate the pathological process of PE [20]. Pregnancy is a complex life process requiring myriads of proteins to provide functional support. The endoplasmic reticulum (ER) has been shown to play an important physiological role in placental development during pregnancy [21]. ERS is a protective stress response of the organism in the face of protein misproduction. ERS is induced when excess unfolded and misfolded proteins accumulate in the lumen of ER, which is seen in the pathogenesis of a variety of pregnancy-associated disorders, such as PE [22], gestational diabetes mellitus [23], fetal growth restriction [24], and spontaneous abortion [25]. Many studies have shown that ERS is involved in PE pathogenesis by mediating genetic factors, placental ischemia and hypoxia, OS, metabolic disorders, impaired angiogenesis and function, and inflammation.

In this review, we will delve into the role of ERS activation in the pathologic progression of PE and discuss targeted inhibition of ERS as a potential treatment option.

## Pathologic progression of PE

Although there is still much controversy regarding the exact etiology of PE, placental dysplasia has been recognized by many scholars as the core pathological basis [17].

The placenta, an important organ during pregnancy, not only serves as a bridge for the exchange of nutrients and materials between the mother and fetus but also as a barrier to provide immune protection to the fetus [17]. During early pregnancy, trophoblast cells from the fetus merge to create chorionic trophoblasts, which, along with the blood vessels in the umbilical cord, form the fundamental functional unit of the placenta and fetal circulation. At the same time, another fraction differentiates into trophoblast outer cells and participates in the remodeling of the vascular wall tissue of the spiral arteries by infiltrating the myometrium. This results in the loss of the smooth muscle musculature of the spiral arterial vasculature, which ensures that the placenta is adequately perfused with blood flow to maintain the nutrient supply to the fetus.

The “six-stage model” proposed by Redman (2014) elaborates the pathologic progression of PE [26]. The initial phase of PE involves the temporary process of embryo implantation, during which the mother exhibits inadequate immune tolerance to paternal genes. The second phase, spanning weeks 8 to 18 of pregnancy, is crucial for placental development, as trophoblast cells start to invade the uterine spiral arteries. It is believed that abnormal placental development triggers PE. Following the abnormal formation of the placenta, the third phase occurs, leading to a stress response. In the fourth phase, which takes place during the middle and later stages of pregnancy, various injury factors from the placenta are released into the mother’s bloodstream. The fifth phase is marked by the emergence of clinical symptoms such as hypertension and proteinuria, allowing for a diagnosis of PE. A small percentage of patients may progress to the sixth phase, characterized by rapid atherosclerosis of the spiral arteries, reduced placental perfusion, and subsequent thrombosis and infarction of the placenta. The majority of significant research on placental insufficiency has been conducted on suprachorionic tissues from the placenta after childbirth, highlighting the harm resulting from an unstable and insufficient maternal blood supply. Consequently, the variations seen between affected and healthy placentas might be wrongly interpreted as direct causes rather than as secondary effects of the progression of the disease. Once impaired placental formation or structural abnormality occurs, it triggers insufficient depth of invasion of trophoblast cells into the muscularis propria and initiates the earliest pathological changes of PE [27, 28]. The impaired vascular recasting of the spiral arteries of the uterus can be reflected by placental hypoperfusion, including placental ischemia, infarction, abnormal placental villous development, and abnormal angiogenic protein levels [27]. The basic pathologic changes in the mother include systemic small-vessel vasospasm and vascular endothelial cell damage, which manifest as multiorgan and multisystem damage [29]. In particular, the placenta of patients with PE is usually associated with increased apoptosis of trophoblast cells and aberrant expression of growth factors, including vascular endothelial growth factor (VEGF) and placental growth factor *(PIGF)* [30, 31], which may be mediated through ERS, inflammatory response, and OS. As a state of imbalance between oxidative and antioxidant effects in vivo, OS is centrally characterized by the overproduction of oxygen free radicals, inducing neutrophil infiltration, increased protease secretion and ultimately tissue damage. More importantly, placental damage leads to the release of cellular debris, metabolic wastes, and soluble factors into the maternal circulation, further exacerbating maternal vascular endothelial cell dysfunction and systemic inflammatory responses [32].

## Association of ERS activation with the pathologic development of PE

### ER and ERS

ER is a reticular system of vesicular and tubular structures interwoven and distributed in the cytoplasm, also called protein folding factories. As an important organelle of eukaryotic cells, ER is categorized into two types: smooth ER and rough ER. Among them, rough ER is related to protein synthesis, processing and transport, while smooth ER is mainly involved in intracellular lipid synthesis and calcium ion storage [33]. Proteins, which play a crucial role in carrying out life functions, can still experience folding issues and errors in the assembly process, even though their synthesis, folding, and modification in the ER are highly regulated. To ensure that protein folding capacity is balanced with demand; cells constantly monitor the amount of misfolded proteins in the ER lumen and initiate corrective programs. Various cellular disorders, including inflammatory responses, energy deprivation, metabolic disorders, and calcium homeostasis imbalances, can lead to unfolded and misfolded protein accumulation in the ER [34, 35]. Under physiological conditions, the processing and translocation of proteins in the ER are in dynamic equilibrium. When the accumulation of misfolded proteins exceeds a critical threshold, the ER is overwhelmed, activating ERS. Once ERS initiates, the cell attempts to correct this state through a downstream signaling pathway triggered by three ER transmembrane proteins, including inositol-requiring enzyme, eukaryotic translation initiation factor 2 alpha kinase 3*(EIF2AK3)*, and activating transcription factor 6, called the unfolded protein response (UPR) [34].

These three ER transmembrane proteins share a common characteristic: they detect the level of misfolded proteins using the same structural domain in the ER lumen. As a result, they change their oligomerization state to transmit signals from the ER lumen to the cytoplasm. Typically, the UPR mitigates ERS through various adaptive responses, including protein synthesis reduction, promotion of protein degradation, and upregulation of transcription of molecular chaperones [34, 35]. Interestingly, to increase protein folding capacity, the UPR also increases the ER membrane area by increasing the biogenesis of genes involved in lipid metabolism, thereby expanding the ER capacity [34]. Overall, the above transcriptional events collaboratively regulate ER stress. If the number of misfolded proteins is successfully reduced, cells will survive the weakened UPR signaling. If persistent UPR signaling still fails to rectify the ERS state, the transmembrane sensors will output pro-apoptotic signals to induce cell death [36, 37]. These ER transmembrane proteins normally bind to heat shock 70 kDa protein 5 *(HSPA5)* to remain inactive. However, when large amounts of unfolded or misfolded proteins accumulate in the ER, *HSPA5* is ectopically released, resulting in activation of ER transmembrane proteins due to conformational changes [33].

*Inositol requiring enzyme 1 (IRE-1)* is an important ERS-sensing molecule, which shears the mRNA of *X-box binding protein 1 (XBP1)* upon activation under stress, resulting in transcription factor activity activation, thereby initiating lipid synthesis and protein degradation to relieve ERS [38]. In addition, IRE-1 activation can lead to RNase activity disruption, which results in mRNA attenuation at the ER membrane, thereby further reducing protein loading to the ER, a process known as regulated *IRE1α*-dependent decay [39]. Studies have shown that *IRE-1* activation inhibits apoptosis, while IRE-1 can interact with *tumor necrosis factor receptor–associated factor 2 (TRAF2)* to promote apoptosis during severe ERS [37]. In addition, *IRE-1* oligomerization has been shown to induce activation or upregulation of many pro-inflammatory proteins, activating inflammatory vesicles and cysteine protease-1 (Caspase-1)-dependent pro-death pathways [40, 41].

Eukaryotic translation initiation factor 2 alpha kinase 3 is a Ser/Thr protein kinase located in the ER, and its activation depends on the phosphorylation of its structural domain. Activated *EIF2AK3* phosphorylates the eukaryotic translation initiation factor 2α *(eIF-2α)* to reduce its activity, thereby decreasing the synthesis and translation of proteins and alleviating the load on the ER [41]. Furthermore, when *eIF-2α* activity was downregulated, the *activating transcription factor 4 (ATF4)* expression could be upregulated. More importantly, *ATF4* negatively feedback-regulates eIF-2α phosphorylation by activating growth arrest and DNA damage-inducible gene-34 *(GADD-34)* and restores ER homeostasis by upregulating *XBP-1* expression. Meanwhile, activated *ATF4* also upregulates the expression of the apoptotic protein, CCAAT enhancer binding protein-homologous protein *(GADDl53)* [38]. High levels of *GADDl53* can inhibit the anti-apoptotic factor B-cell lymphoma-2 (Bcl-2) expression, thereby promoting apoptosis [42].

*ATF6*, as a transcription factor, unlike the other two transmembrane proteins, is not involved in the pro-apoptotic signaling of the UPR [43], which is released from membrane proteins on the ER membrane under stress, enters the nucleus, and upregulates the transcription of genes, such as *HSPA5* and *XBP-1*, by translocation to the Golgi apparatus and cleavage by proteases, which increases the protein-folding capacity of the ER and promotes cell survival [36].

### The dual role of ERS in PE progression

#### Moderate ERS is essential for early placental development

**Figure 1:**
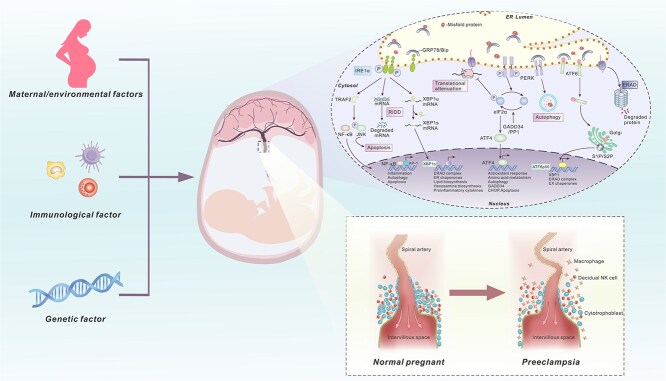
Genetic, environmental, and immune risk factors activate ERS signaling, which is classified as PERK, IRE1α, and ATF6. They respond to protein folding stress and stress responses in the placenta by regulating target gene transcription or inducing apoptosis. Once continuously disturbed by ERS signaling, the placenta undergoes developmental disorders characterized by apoptosis of trophoblast cells, inhibition of trophoblast differentiation, and restriction of recasting of uterine spiral arteries as the main pathological features (ERS: endoplasmic reticulum stress).

ERS is not only a stress defense response of the organism but also plays an important role in early placental development (Figure 1). Heat shock 70 kDa protein 5 *(HSPA5)*, an ER-resident protein that is a major triggering device of the UPR [44], is expressed on the cell surface of trophoblast cells and may play a key role in the formation of syncytial trophoblast cells [44]. Although *EIF2AK3*, *ATF6,* and *IRE1* act as downstream signaling for UPR, they have different functions in vivo. Studies have demonstrated that *EIF2AK3* is involved in various placental development stages by regulating trophoblast differentiation [45]. The two isoforms, *ATF6α* and *ATF6β*, are widely expressed in mammals, and their simultaneous knockdown early in the development process affects embryo survival, although knockdown of one of the two genes does not result in developmental abnormalities [46, 47]. Similarly, IRE1α is ubiquitously expressed in fetal and adult mice, which is essential for mammalian developmental processes [48]. Previous studies have found that *IRE1α* deficiency exacerbates ERS and leads to severe placental dysfunction and embryonic death [49]. Placental trophoblasts of *IRE1α* gene-deficient knockout mice exhibit significant structural disruption compared to wild-type mice, severely affecting maternal–embryonic nutrient exchange. It is well established that VEGF-A is not only a catalyst for angiogenesis but also regulates the development of placental villous tissue, and activation of *IRE1α* is required for the expression of VEGF-A by trophoblast cells [49]. If the UPR pathway is not activated, the VEGF-A level is insufficient to complete angiogenesis, leading to abnormal placental development and impaired exchange of substances between the mother and fetus, strongly validating the UPR signaling importance for placental development [50]. Two other downstream targets associated with the UPR, *ATF6* [51] and *EIF2AK3* [52], have also been reported to be involved in the VEGF-A regulation. It has been found that the pathologic features of IRE1α-deficient placentas also include reduced trophoblast proliferation and poor ER development in the trophoblast [53]. Importantly, reduced trophoblast proliferation and trophoblast ER dysplasia appear to arise from the ability of *IRE1α* to enhance RNAase activity and recognize and disassemble mRNAs with stem-loop structures to reduce ERS and may also be a contributing factor to embryonic death [51]. In conclusion, an appropriate ERS response is essential for placental development by improving trophoblast cells’ proliferative activity and secretory function. However, hyperactivation of the ERS pathway can lead to apoptosis, adversely affecting placental formation and pregnancy outcome.

#### ERS affects trophoblast differentiation during PE progression

Trophoblasts are a major component of the developing placenta, and their aberrant differentiation is a central pathologic basis for placenta-related pregnancy complications [20, 54, 55]. Trophoblasts in the human placenta are classified into three different subtypes including trophoblasts (CTB), extravillous trophoblasts (EVT), and syncytiotrophoblasts (STB) with different anatomical locations, histological structures, and physiological functions, respectively [56, 57]. During trophoblast differentiation, CTB cells stimulate stem cell activity and differentiate into the STB cell layer by sequential fusion or undergo epithelial–mesenchymal transition to form EVT cells [58]. STBs are the outer layer of the placental villi that act as a regulatory hub for nutrient exchange between the mother and the fetus and secrete hormones and proteins that protect the fetus from pathogenic bacteria [59]. EVTs initiate infiltration and invasion of the endometrium, myometrium, and spiral arteries from the site of the encapsulated embryonic sac, establish uteroplacental circulation, and ensure successful placental encapsulation [54, 60]. Evidence suggests hypoxia, inflammation, genetic factors, OS, and metabolic disorders promote sustained ER overload or molecular chaperone dysfunction [61–65]. Many studies have shown that trophoblast differentiation dysfunction induces the above pathology and leads to adverse pregnancy outcomes in PE [54, 66]. The PE development is associated with an ERS-mediated process of abnormal differentiation of trophoblasts, ultimately leading to pathological changes in placental structure and function. A study of placental histology in patients with PE demonstrated focal syncytial trophoblastic necrosis, rough ER enlargement, and reduced trophoblastic activity [50]. A study observed that blastocysts of mice with a chronic ERS model exhibited increased expression of ERS-related markers and also showed reduced trophoblast numbers and developmental delay compared to blastocysts of wild-type mice [25]. Interestingly, when ERS-targeted inhibitors were added, placental trophoblast subtype abundance was successfully restored, and embryo mortality was significantly reduced [25]. Another investigation found that ERS may affect the differentiation of mouse trophoblast stem cells into other subtypes and may increase the incidence of early miscarriage [25]. High levels of homocysteine have been reported [63] to activate the UPR in mouse blastocysts, decreasing the trophoblast count and inducing their premature differentiation, which can lead to early pregnancy abortion [67]. This pathologic change can also be partially reversed by the ERS inhibitor tauroursodeoxycholic acid (TUDCA). Furthermore, puzzlingly, reduced mRNA and protein expression of the ERS marker *HSPA5* was observed in trophoblasts of women with PE [68]. Indeed, it has been described that decreasing *HSPA5* expression by siRNA treatment decreases the STB generation in vitro. This contradicts the previously validated statement between ERS and trophoblast differentiation in PE [69]. This difference may be attributed to different subtypes of trophoblasts having various degrees of adaptive response to ERS. In some cases, the dual effect of ERS on trophoblast differentiation may be related to different stimuli for intervention [68, 70, 71].

Overall, both abnormal trophoblast differentiation and placental structure may be negatively regulated by ERS, as reflected by marked thinning and vacuolization of the STBs, increased basement membrane folding, and regional STB necrosis [72]. However, the potential mechanism by which ERS affects PE pathological progression by interfering with trophoblast differentiation remains to be investigated further.

#### ERS induces trophoblast apoptosis in the development of PE pathology

The close link between aberrant trophoblast apoptosis and the pathogenesis of placenta-associated diseases, including PE, has been confirmed by many studies. In addition, ERS is an important molecular mediator linking between trophoblast apoptosis and PE [73, 74]. As a bridge between the fetus and the mother, the placenta is constantly under a high load of protein synthesis and folding to accommodate its complex metabolic functions. The accumulation of misfolded proteins in the ER lumen of trophoblasts can lead to the development of ERS [75]. Despite the efforts of the body to inhibit the transcription of misfolded proteins and correct them, the irreversible accumulation of misfolded proteins leads to apoptosis in trophoblasts [75–77]. Trophoblasts triggered severe apoptosis and ERS in an in vitro simulated hypoxic environment [78]. In addition, increasing methylation of ER disulfide oxidase 1 alpha *(ERO1α)* feedback activated the ERS and induced an increased number of apoptotic cells in the trophoblast of rat placenta [79]. There is much clinical evidence of increased apoptosis of placental trophoblasts in PE pregnancies compared to normal pregnancies, as confirmed by immunohistochemical pathologic changes [80]. In trophoblastic tissues of PE, ERS and apoptosis are concomitant, and instead, ERS promotes trophoblastic apoptosis and PE progression [78]. In other words, *XBP1* targets miR-148a and initiates trophoblast apoptosis, and inhibiting *XBP1* activity significantly reduces trophoblast apoptosis and attenuates preterm labor [81]. Similarly, activation of the *EIF2AK3/ATF4/GADDl53* pathway by perfluorooctanoic acid in the early UPR is an important trigger of apoptosis in PE trophoblasts [82]. Specifically, *GADDl53* is a transcription factor homologous protein that signals trophoblast apoptosis with cellular stress responses, particularly ERS. Also, the *GADDl53*, Caspase-12, and c-Jun N-terminal kinase (JNK) signaling pathways are significantly activated in PE trophoblasts. In a study, metallic lead exposure induced apoptosis in placental trophoblasts through ERS and Caspase-12 activation [83]. Placental trophoblast apoptosis is closely related to *GADDl53* expression, and inhibition of *GADDl53* overexpression contributes to normal placental development. Moderate apoptosis of trophoblasts is a part of the physiological renewal metabolic program of placental tissues, whereas excessive, aberrant apoptosis is an important feature of the PE progression. Overall, ERS-induced trophoblast apoptosis is strongly associated with pathological changes in PE, but the specific molecular mechanisms involved are unclear.

## Possible mechanisms of ERS-mediated pathologic progression of PE

### Placental hypoxia promotes PE by mediating ERS

The oxygen levels in the placental microenvironment fluctuate significantly during pregnancy, as it is a complex physiological process [84]. These oxygen concentrations are vital for regulating the development of organs and tissues, as they help coordinate the interactions among various trophoblast cells and molecules that influence placental growth. Research involving various mammals has shown that blastocyst implantation can take place in the uterus at oxygen levels as low as 2% to 3% or in conditions with an average partial pressure of oxygen ranging from 15 to 20 mmHg [85, 86]. This low-oxygen environment supports the development of preimplantation embryos and keeps their metabolic activity low, which helps decrease the generation of reactive oxygen species (ROS). Additionally, the placenta exists in a naturally low-oxygen setting during the early stages of pregnancy, despite the fact that several important processes for placental development take place during this time, such as the implantation of the blastocyst, the invasion of trophoblasts, and the remodeling of spiral arteries [87]. As pregnancy advances, the reduction in placental oxygen levels triggers the activation of several oxygen-sensitive signaling pathways, such as hypoxia-inducible factor (HIF) [88], *mammalian target of rapamycin (mTOR)* [89], and the UPR [90]. These pathways subsequently influence gene expression, metabolic balance, and cell survival. Conversely, defects in these pathways can result in problems with the placenta. When the placenta experiences hypoxia, the processes of trophoblast cell invasion, proliferation, differentiation, and apoptosis become irregular. This can cause inadequate trophoblast invasion, restricted blood flow, and placental dysfunction, ultimately resulting in various pregnancy complications such as PE and significantly impacting pregnancy outcomes.

**Figure 2:**
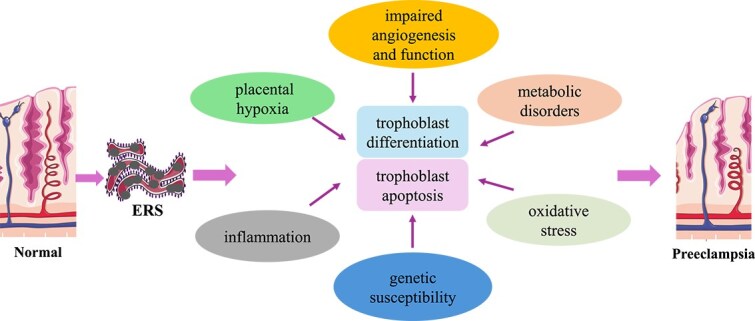
ERS is associated with the pathomechanism of PE. ERS participates in the process of pathological changes in PE by mediating placental hypoxia, inflammation, oxidative stress, genetic susceptibility, metabolic disorders, and impaired angiogenesis and function (ERS: endoplasmic reticulum stress; PE: preeclampsia).

Small-vessel spasms and impaired recasting of the uterine spiral arteries are central pathologic features of PE and major predisposing factors for placental hypoxia (Figure 2). Oxygen is a terminal receptor for the mitochondrial electron transport chain and a key substrate for ER protein synthesis and folding. Low oxygen levels have been shown to disrupt mitochondrial reactive oxygen species (ROS) homeostasis and induce ERS. It has been demonstrated that hypoxia leads to excessive ROS overflow and triggers placental mitochondrial damage by altering the protein structure and activity, such as oxidizing cysteine sulfhydryl group (Cys-SH) to cysteine sulfonic acid (Cys-SOH) [91]. Mennerich et al. found that ERS can regulate the response to hypoxia-inducible factor-1α (HIF-1α) and hypoxia-inducible factor-2α (HIF-2α) through the *glycogen synthase kinase-3 beta (GSK3β)/β*-transducin repeat-containing proteins (β-TrCP) axis in response to hypoxia [92]. In addition, HIFs play a key role in PE by responding to placental hypoxic stimuli. Hypoxia stimulates ROS release from the mitochondrial complex, which stabilizes HIFs’ activity [93]. HIFs inhibit mitochondrial metabolic activity by affecting the expression of key enzymes in the tricarboxylic acid cycle, thereby interfering with the normal folding of proteins in the ER [94]. Interestingly, HIF-1α and *GADDl53* were simultaneously increased in PE placental samples, suggesting that HIF-1α may be an important transit structure between hypoxia and UPR [95]. Previous evidence suggests that severe placental hypoxia activates the *EIF2AK3* pathway of the UPR, leading to UPR and HIF signaling [96]. This implies that ERS can further contribute to hypoxic response. Notably, placental hypoxia was confirmed in a PE rat model [97] and was accompanied by a large number of misfolded proteins in trophoblasts [98]. There is also substantial evidence that significant elevation of many ERS markers, such as *HSPA5*, *p-EIF2AK3*, *ATF4, ATF6*, and *GADDl53*, are observed in placental tissue in PE [95, 99–101]. It was demonstrated that in PE placentas, hypoxia may increase *ATF6* expression and nuclear translocation and lead to UPR signaling pathway activation and morphological alterations of ER structures during ERS [102]. Meanwhile, transmission electron microscopic observation further showed that the ER cristae of the hypoxic placenta in PE were significantly dilated compared to the normal placenta [103]. In addition, another study showed that UPR signaling-related proteins, including *ATF6* and *HSPA5*, were also upregulated in PE placental hypoxia [104]. In summary, the impact of hypoxia on trophoblasts is a complicated and difficult area of research, and many existing in vitro cellular experiments have certain limitations. Future investigations into the actual influence of oxygen partial pressure on placental trophoblast development in PE in vitro will require validation through in vivo studies.

### ERS interacts with OS in the PE progression

Numerous studies have shown that OS is associated with PE progression [105, 106]. Mitochondria are one of the major organelles that sense oxygen and produce energy. The most prominent pathological feature of OS is large amounts of ROS accumulation in the mitochondria, ER, and other membranous structures. Many studies have described elevated OS markers, such as ROS, in the placental tissue of women with PE [107, 108]. Peroxynitrite is a highly radical-active oxidant that induces nitrosylation of protein tyrosine residues, which is thought to be a marker of excessive intracellular ROS accumulation. Notably, the ROS and peroxynitrite concentrations in rat placenta increased with the degree of PE [109]. In addition, OS has been associated with trophoblast apoptosis, inhibition of trophoblast differentiation, inflammation, and placental dysfunction in PE [72, 110]. ROS is a double-edged sword, and studies have shown that an appropriate amount of ROS can act as a second messenger, regulating various physiological activities of cells [111]. Due to its high reactivity, ROS overaccumulation may impair the processes of oxidative phosphorylation and oxidative protein folding in placental trophoblasts. Signaling communication between mitochondria and ER plays an important role in placental function. ERS may catalyze OS and promote PE progression by activating inositol 1,4,5-trisphosphate receptor, NADPH oxidase 4, and promoting the formation of disulfide bonds [112]. Specifically, much evidence suggests communication between ERS and OS in the PE pathomechanism. Elevated levels of ROS can impair the ER redox homeostasis, thereby hindering protein synthesis and folding. In addition, ROS can induce calcium ion disturbances by increasing the sensitivity of calcium channels in the ER membrane, reinforcing ERS [113].

### Metabolic disorders affect PE through ERS activation

As a specific hypermetabolic state, metabolic disorders during pregnancy play a key role in the PE pathogenesis [114]. Obesity and abnormal weight gain during pregnancy are high-risk factors for PE [115]. One study showed that obese pregnant women were far more likely to develop PE than those with normal body mass [114]. Metabolic abnormalities, including insulin resistance, elevated glucose, leptin, and lipid levels, usually characterize obesity [116].

Remarkably, high levels of circulating cholesterol and uric acid are also associated with increased placental inflammation and dysfunction [117]. In addition, obesity induces inflammatory cytokine secretion and hypoxia to interfere with placental metabolic functions [118, 119]. ERS markers were significantly elevated in the placental STB and CTB in obese pregnant women compared to controls and were accompanied by PE [120]. In placental trophoblasts, palmitate upregulated protein and gene expression of *GADDl53*, *HSPA5*, and *XBP1* through ERS stimulation, along with a significant increase in apoptosis [121, 122]. Appropriately elevated maternal cholesterol during pregnancy contributes to the normal physiologic development of the fetus. When cholesterol levels are abnormally high, cholesterol accumulation in adipose tissue disrupts protein folding through ERS signaling and also impairs placental vascular endothelial function [120, 123]. Many studies have demonstrated that hyperglycemia during pregnancy is a potential risk factor for PE [124, 125]. Hyperglycemia may be a key factor linking the pathomechanisms between ERS and PE. A proteomics study also confirmed the enrichment of 192 acetylated proteins associated with hyperglycemia during pregnancy and PE. Interestingly, these proteins were mainly enriched in the ERS and iron death signaling pathways [126]. Specifically, hyperglycemia may promote ectopic glucose transporter protein-3 (GLUT3) expression through *AMPK* activity activation and increase glycolytic flux, which ultimately impairs trophoblast invasiveness and exacerbates PE symptoms [127]. The ERS state of the PE placenta has been reported not only to promote the secretion of misglycosylated proteins but also to lead to maladaptation of maternal glucose metabolism [128]. In addition, dysregulated glucose transporter protein-1 may be involved in the PE pathogenesis through ERS-impaired metamorphosis [129]. Interestingly, glucose deprivation causes particulate shedding of STB cells and exacerbates PE [130]. Although ERS has a negative effect on placental glucose and lipid metabolism, the specific mechanisms underlying the association of ERS with pathologic changes in PE remain to be further investigated.

### Impaired ERS-induced angiogenesis and function exacerbate PE

Impaired placental angiogenesis is recognized as a key factor in the development of PE. In vitro, co-culture of serum from pregnant women with PE with placental tissues revealed a significant increase in the expression of key targets of ERS signaling such as *HSPA5*, *GADDL53*, and *GADD34* [131]. In addition, ERS-induced trophoblast apoptosis promotes excessive shedding of STB microparticles (STBM) into the maternal circulation, a pathologic feature of early PE. Previous studies have shown that *PIGF*, soluble fms-like tyrosine kinase 1 (sFlt-1), and soluble endoglin (sEng) vascular-generating factors are involved in the development of PE [132]. VEGF, *PIGF*, and fibroblast growth factor 2 (FGF2), as key factors regulating the placental angiogenesis, are mediators of ERS and PE. Specifically, downregulation of *PIGF* proteins was associated with nuclear localization of the UPR transcription factors *ATF4, ATF6α*, and *ATF6β* in the placental STB of patients with PE [100]. At the same time, ERS activation in the placenta leads to a decrease in the secretion of circulating *PIGF* in the mother, which induces impaired angiogenesis in the PE placenta [100]. In addition, activation of the ERS branch *EIF2AK3* markedly upregulates the transcription of the *VEGF* and *FGF2* genes, and binding of human sFLT1 to circulating VEGF and *PIGF* appears to impair the angiogenic process and leads to impaired placental trophoblast invasion [131]. The discovery that stromal cell-derived factor 2 (*SDF2*), a component of the Hsp90-eNOS complex in human endothelial cells, is involved in regulating placental angiogenesis and also initiates placental UPR signaling lays the groundwork for investigating the connection between ERS and the development of PE [133]. In addition, some cytokines, such as transforming growth factor β (*TGFβ*), HIF-1α, and type 1 angiotensin II, are also involved in the placental angiogenesis regulation during PE progression. However, further study is still required on whether ERS mediates them.

### Significance of ERS in inflammatory and immune responses in PE

A systemic inflammatory response characterizes pregnancy, especially when combined with PE. The expression of inflammatory factors in blood circulation is significantly increased in patients with PE. Evidence shows that ERS may act synergistically with inflammation, contributing to the PE progression. In addition, it has been reported that the expression of inflammatory factors, such as tumor necrosis factor-α (TNF-α), interleukin-6 (IL-6), interleukin-1 β (IL-1β), interferon-gamma (IFN-γ), TGFβ1, monocyte chemotactic protein-1 (MCP-1), and *PIGF*, were expressed at a significantly higher level in the STB of women with PE than in normal pregnant women, and were also accompanied by activation of ERS signaling [134–136]. Several studies have shown that inflammatory factors, such as TNF-α, IL-6, and IL-1β, are arthritic factors that mediate ERS activation and promote apoptosis in PE trophoblasts [110, 137]. Inflammatory factors play critical bridging functions in the ERS and PE placental inflammatory response. Specifically, TNF-α induces endothelial and trophoblastic dysfunction with concomitant activation of ERS and inflammatory reactions by stimulating intercellular cell adhesion molecule-1 (ICAM-1) expression in the placenta. Meanwhile, ILs are involved in PE development by mediating the differentiation cluster 81 to promote helper T cell 17 (Th17) differentiation in response to ERS signaling and disrupting the maternal inflammatory and immune-tolerant environment [138, 139].

IL-11, regulated by IL-1β, is associated with trophoblast and placental function and induces pathological PE features in mice [140–142]. Elevated expression of *HSPA5*, a major regulator of ER stress, was found after treatment of early human placental explants with 100 ng/mL of IL-11 for 24 h. These data suggest that IL11, a risk predictor for obstetric complications such as PE, may act by triggering ERS in the placenta. ERS also interferes with placental vascular endothelial function through an inflammatory response and promotes PE progression [143]. Angiogenic factors, such as sFlt-1 and VEGF, act as potent ERS signaling mediators and enhance the inflammatory manifestations of the placenta in pregnant women with PE [144]. A proteomics-related study showed that *chemokine ligand 20 (CCL20)* was significantly differentially expressed in the serum of women with PE compared to normal women [145]. Notably, various cytokines, such as VEGF, sFlt-1, IL-1β, IL-17, TNF-α, and *EIF2AK3*, as well as *ATF6* targets of the UPR signaling pathway, closely interacted with *CCL20* [146–149]. ERS can be activated or induced by OS, calcium dysregulation, NLR family pyrin domain-containing protein 3 *(NLRP3)* inflammatory vesicles, and nuclear factor kappa B *(NF-κB)* placental inflammatory response. Recent evidence suggests that ERS is involved in the inflammatory response in the placenta of patients with PE by mediating the *NLRP3* inflammatory vesicle activation [150] by thioredoxin-interacting protein *(TXNIP)* [151]. It is well established that *NF-κB* acts as a nuclear transcription factor that enters the nucleus and promotes the transcription of multiple pro-inflammatory mediators during the placental inflammatory response. This occurs naturally during a normal pregnancy but is especially high in PE. It has been reported that ERS and UPR signaling upregulation may impair TNF-α and calcium homeostasis, cause dysregulation-induced apoptosis in placental trophoblasts at an early stage, and promote PE progression [152, 153]. In addition, 4-phenylbutyric acid (4-PBA), an inhibitor of ERS, alleviated hypoxia- and inflammation-induced apoptosis in PE trophoblasts by inhibiting the *EIF2AK3*/*ATF4*/*GADDl53* pathway [137].

### ERS and calcium homeostasis involved in the PE regulation

ERS has been shown to cause calcium homeostasis imbalance, and these two are important factors affecting placental function in PE [154]. Calcium is an important secondary messenger involved in many intracellular pathophysiological processes, such as apoptosis, neural signaling, enzyme, and hormone secretion, and is critical for placental development and prenatal disease planning [155]. Calcium ions are transported across the placenta at 140 mg/kg/d, including placental cotyledon-mediated paracellular diffusion and STB-mediated epithelial transport. The elevated calcium transport capacity of the syncytiotrophoblast (STB) in the placenta supports proper fetal growth and development, particularly in the formation of bones and teeth as well as in the regulation of neuromuscular functions. Intracellular calcium is primarily stored in the ER. ERS-triggered calcium inward flow possesses different pathways and mechanisms from the high levels of intracellular calcium transport in the placenta. In the case of severe or prolonged ERS, Ca^2+^ is released from the ER into the mitochondria, subsequently triggering a series of downstream signaling. Studies in pregnant sheep have confirmed that calcium overload and dysregulation of spontaneous transient outward currents produced by hypoxic stimuli provided to isolated uterine arteries promote placental vascular dysfunction by mediating ERS. Unfortunately, this study was missing data on clinical signs following elevated uterine artery pressure in sheep [152]. Encouragingly, the disruption of calcium balance and poor adaptation of uterine blood vessels caused by hypoxia showed significant improvement following the inhibition of ERS. Nonetheless, numerous studies still face various limitations, and there is a notable scarcity of evidence from in vivo research [152, 156]. In addition, ERS-induced dysregulation of placental calcium homeostasis is mediated through calcium channels, calcium-binding proteins, ATP synthase, and calcium ion pumps. In vitro culture experiments on primary STBs revealed that calcium transport was significantly attenuated in STBs of PE [156]. In addition, the protein expression levels of calcium channels and calcium-binding proteins, such as *TRPV5, TRPV6, CaBP-9 K*, and *CaBP-28 K*, were significantly reduced in PE STBs, in contrast to the upregulation of the mRNA expression of sarcoplasmic reticulum calcium-conjugating ATPases, such as *SERCA1* and *SERCA2* [156]. The results of two large-scale clinical trials showed that calcium supplementation reduced the PE risk in pregnant women with calcium deficiency or high-risk pregnancy factors [157, 158]. Unfortunately, however, some randomized trials have also reported no significant benefit of calcium supplementation in reducing the incidence and severity of PE [159]. These studies seem to reveal that PE is associated with an imbalance in calcium homeostasis rather than a calcium deficiency, which side-steps the validation of why there is a great deal of uncertainty about the efficacy of calcium supplementation. The specific molecular mechanism of ERS-mediated calcium homeostasis imbalance involved in the PE pathological progression still needs further investigation.

### Interaction between ERS and genetic susceptibility in PE progression

It is well established that a family history of PE and some susceptibility genes are high-risk factors for PE development. It was shown that the IL-17A polymorphism (rs2275913), which is associated with the expression of inflammatory factors, was associated with the PE pathogenesis in the Chinese regions [160]. The pentraxin 3 (*PTX3*) gene encodes an inflammatory response protein that has been shown to mediate mitochondrial dysfunction in oocytes in conjunction with ERS signaling in obese mice [161]. Plasma *PTX3* protein levels were significantly higher in PE patients than in controls. Meanwhile, receiver operating characteristic analysis showed that a 3′-untranslated region polymorphism of the *PTX3* was also closely associated with PE risk [162]. In addition, the expression of *PTPRK*, a gene localized in placental trophoblasts, has been verified to be associated with the PE development [163]. PTPRK has been shown to be associated with invasive viability of trophoblast cells, which, in turn, is regulated by activation of the ERS-related *MAPK* signaling pathway [164, 165]. Unfortunately, molecular evidence for the association of PTPRK with ERS is still relatively lacking. The killer cell immunoglobulin-like receptor 2DL4 (*KIR2DL4*) gene, which functions as an immune tolerance at the maternal–fetal interface, is highly associated with PE development [166]. One study found a genetic association between the rs1126579 polymorphism in the CXCR2 and an increased PE risk. Myeloid-derived suppressor cells (MDSC) are a diverse collection of myeloid cells that have immunosuppressive properties, primarily by inducing ERS and utilizing suppressor-related enzymes. Interestingly, *KIR2DL4* and *CXCR2* play a role in the immune tolerance function of MDSC, particularly in managing the maternal-fetal interface throughout pregnancy. Furthermore, while genetic screening and gene localization cloning techniques have been utilized in the examination of potential genes associated with PE, a limited number of studies focus on ERS signaling targets. Additionally, there is a lack of a well-defined consensus system for identifying genes that make individuals susceptible to PE.

## ERS as a potential target for PE treatment

Due to the lack of research and limitations in understanding specific molecular mechanisms, there is no effective clinical approach to treat PE other than delivery. In recent years, the key role played by ERS in PE pathogenesis has been increasingly emphasized, and it has emerged as a promising therapeutic target for PE as compounds with “molecular chaperone” activity, 4-PBA and TUDCA have been shown to reduce ERS-associated damage by blocking the UPR activation. 4-PBA is a short-chain fatty acid with histone deacetylase activity. Consistently, 4-PBA alleviates hypoxia-induced trophoblast apoptosis and autophagy by inhibiting the *EIF2AK3*/*ATF-4/GADDl53* pathway in vitro [137]. TUDCA, a bile acid derivative, is approved for safe use in treating intrahepatic cholestasis during pregnancy. In addition, TUDCA has been shown to improve uterine arterial vascular function and adverse pregnancy outcomes in complicated pregnancies by inhibiting ERS [167]. *ATF4* inhibition was shown to alleviate apoptosis in placental trophoblasts and improve the PE progression [131]. In addition, as a key transcription factor, the *XBP1s* nuclear translocation can alleviate ERS severity during pregnancy when disrupted by knockdown [168]. Due to the specificity of metabolism and fetal development during pregnancy, targeted inhibitors of the UPR signaling pathway may have potential pharmacological toxicity and teratogenic effects. Despite the significant therapeutic effects, the application of ERS-targeted inhibitors needs to be further explored.

Due to their lower pharmacological toxicity, natural compounds derived from herbal medicines for ERS are constantly being explored and are expected to become alternative therapies for PE. Salvianolic acid A is a water-soluble phenolic substance extracted from the traditional Chinese medicine *Salvia miltiorrhiza*, which has antioxidant, antimicrobial, and anti-inflammatory pharmacological effects. Studies have shown that Salvinorin A exerts antioxidant and vasoprotective effects by inhibiting the degree of ERS in human brain microvascular endothelial cells (HBMECs) and decreasing their levels of ROS and calcium expression, implying that it has great potential for the treatment of PE [169, 170]. Baicalein is a bioactive flavonoid extracted from the roots of Huang Cen, containing antioxidants, regulating lipid metabolism, and having anti-apoptotic effects. A model of PE in pregnant rats was constructed using NG-nitro-L-arginine methyl ester (L-NAME), and the treatment groups were given different concentrations of 50, 100, and 150 mg/kg·d of xanthoside for 20 days. The results showed that baicalein effectively reduced blood pressure and restored kidney and liver function in PE rats [171]. Baicalein reduces ERS caused by high glucose levels by increasing the expression of the *silencing regulator protein 3 (SIRT3)*, which helps preserve the normal function of retinal microvascular endothelial cells [172]. In addition, gallic acid ester is a natural compound extracted from green tea, and clinical trials have shown that gallate ester improves the therapeutic efficacy of antihypertensive medications for severe PE without causing additional adverse effects [173]. In addition, gallate has demonstrated antioxidant function through activation of eNOS, thereby ameliorating vascular endothelial cell dysfunction in PE [174]. Also, gallic acid attenuated ischemia–reperfusion injury in rat testis by inhibiting ERS [175]. Atractylenolide attenuated apoptosis and OS in human trophoblasts in PE through *MAPK/ERK* signaling pathway activation [176]. In addition, atractylenolide alleviates in vitro matured porcine oocyte damage by attenuating *Clostridium difficile*–induced ERS. Network pharmacology and molecular docking results demonstrated that resveratrol could regulate angiogenesis and anti-inflammation by modulating the AGE-RAGE and HIF-1 signaling pathways and could be a valuable drug candidate for PE treatment [177]. Meanwhile, resveratrol exerts neuroprotective functions in injured spinal cord tissues by modulating ERS and inflammation [178]. The natural products of many herbal medicines have demonstrated great pharmacological potential in modulating ERS and alleviating pathological changes in PE. Further studies are still needed to investigate the molecular mechanisms of TCM monomers in targeting ERS for PE in the future.

Exosomes are discoidal vesicles encasing RNA, DNA, proteins, or lipids, extensively involved in cell proliferation, migration, and intercellular communication. Currently, exosomes have demonstrated potential therapeutic value in PE [179]. This involves enhancing the function of placental trophoblasts and endothelial cells, encouraging the formation of new blood vessels, and providing a regulatory immune response along with anti-inflammatory effects. Specifically, human umbilical cord MSC (hUCMSC)–derived exosomes were shown to alleviate the pathological progression of PE in mice by activating Wnt/β-catenin signaling [180]. In addition, hUCMSC-derived exosomes had a protective effect on placental morphology and angiogenesis in PE rats [181]. Furthermore, exosomes derived from human placental microvascular endothelial cells regulate the proliferative and invasive functions of the placental trophoblast in PE by a mechanism related to targeting IGF1 signaling [182]. Exosomes obtained from adipose-derived stem cells reduce liver ischemia–reperfusion injury by suppressing ERS and inflammatory reactions. Furthermore, exosomes from hepatocellular carcinoma cells boost the antitumor immune response of dendritic cells during instances of ERS. However, the potential application of exosomes for PE treatment by mediating ERS signaling is currently unknown, and the specific molecular mechanisms still need to be further investigated.

## Discussion and prospects

The pathogenesis of PE is complex and varied, and the risks to the pregnant mother and the fetus are enormous. Unfortunately, there are no satisfactory strategies for PE treatment other than immediate delivery. ER plays an important role in protein synthesis, translocation, folding, and signaling in the trophoblasts of the placenta. Research on the role of ERS in PE pathogenesis is still in the early stages, and many studies support this view. This article summarizes what is currently available about ERS intervention in PE and some potential therapeutic approaches targeting ERS signaling to alleviate PE. The current study suggests that persistent or severely activated ERS signaling promotes PE progression by mediating genetic susceptibility, placental hypoxia, OS, metabolic disorders, impaired angiogenesis and function, and inflammatory responses. However, ERS under physiological conditions is essential for developing placental trophoblasts. Thus, there are many questions regarding the interactions between ERS and PE. In recent studies, direct evidence of ERS activation as a key component of PE pathophysiology is still relatively lacking. Moreover, it is worth investigating whether the three branches of UPR in placental tissues coordinate their actions during the pathologic process of PE. Additionally, it is crucial to determine whether the activation of these branches follows a logical temporal and sequential order in the organization of the placenta. Furthermore, since the placenta is a complex organ, it is important to understand whether different cell types within the placenta have varying thresholds for activation of ERS caused by various factors. Addressing these questions is essential for advancing future therapeutic approaches for PE. It cannot be ignored that moderate stress is the organism’s self-defense response in the face of pathological factors. Unrestrained suppression or eradication of ERS is undesirable, particularly during the intricate physiological process of pregnancy. While there are advantages to targeting ERS inhibitors in various diseases, it remains unclear whether these inhibitors have any permanent adverse effects on the placenta and the developing fetus. Further research is desired to enhance our understanding of the relationship between ERS and PE.

## Conclusion

Studies focusing on the associations between ERS and PE pathogenesis are still in their inception. We summarize the current relevance of ERS influencing the PE progression and assess the potential of targeting ERS for PE treatment. Our work aims to contribute to the development of studies exploring the association between ERS and PE pathogenesis, and we also hope that it can aid in developing therapeutic drugs for PE that target ERS.

## Data Availability

The data used to support the findings of this study are included within the article.
